# Comparisons of mutation rate variation at genome-wide microsatellites: evolutionary insights from two cultivated rice and their wild relatives

**DOI:** 10.1186/1471-2148-8-11

**Published:** 2008-01-16

**Authors:** Li-zhi Gao, Hongyan Xu

**Affiliations:** 1 Plant Germplasm and Genomics Center, Kunming Institute of Botany, The Chinese Academy of Sciences, Kunming 650204, P. R. China; 2 Department of Biostatistics, Medical College of Georgia, 1120 15th St., Augusta, GA 30912, USA

## Abstract

**Background:**

Mutation rate (μ) per generation per locus is an important parameter in the models of population genetics. Studies on mutation rate and its variation are of significance to elucidate the extent and distribution of genetic variation, further infer evolutionary relationships among closely related species, and deeply understand genetic variation of genomes. However, patterns of rate variation of microsatellite loci are still poorly understood in plant species. Furthermore, how their mutation rates vary in di-, tri-, and tetra-nucleotide repeats within the species is largely uninvestigated across related plant genomes.

**Results:**

Genome-wide variation of mutation rates was first investigated by means of the composite population parameter θ (θ = 4Nμ, where N is the effective population size and μ is the mutation rate per locus per generation) in four subspecies of Asian cultivated rice *O. sativa *and its three related species, *O. rufipogon*, *O. glaberrima*, and *O. officinalis*. On the basis of three data sets of microsatellite allele frequencies throughout the genome, population mutation rate (***θ***) was estimated for each locus. Our results reveal that the variation of population mutation rates at microsatellites within each studied species or subspecies of cultivated rice can be approximated with a gamma distribution. The mean population mutation rates of microsatellites do not significantly differ in motifs of di-, tri-, and tetra-nucleotide repeats for the studied rice species. The shape parameter was also estimated for each subspecies of rice as well as other related rice species. Of them, different subspecies of *O. sativa *possesses similar shape parameters (***α***) of the gamma distribution, while other species extensively vary in their population mutation rates.

**Conclusion:**

Through the analysis of genome-wide microsatellite data, the population mutation rate can be approximately fitted with a gamma distribution in most of the studied species. In general, different population histories occurred along different lineages may result in the observed variation of population mutation rates at microsatellites among the studied *Oryza *species.

## Background

Microsatellites are composed of tandemly repeated, simple DNA sequence motifs of 1–6 nucleotide bases in length. These loci are ubiquitously found throughout both prokaryotic and eukaryotic genomes and typically are highly polymorphic within species and populations. As such, microsatellites have become one of the most popular types of molecular markers and widely employed to study population structure of a diverse range of organisms [1,2], reconstruction of evolutionary history [3], parentage and relatedness analysis [4], and natural selection [5]. Despite the widespread application of microsatellites, their evolutionary dynamics across loci within the species and across related species are yet to be well understood. A powerful approach to study the mutational dynamics of microsatellites during the evolutionary process is to investigate their variation of mutation rates. The development of genomic technology provides an unprecedented opportunity to generate increasing microsatellite data from model organisms and their related species on a genome-wide scale. There are growing interests in using such microsatellite data, as they make it possible to take mutation rate variation into consideration by analyzing multiple loci across related species rather than a single locus from a single species.

Mutation rate (μ) per generation per locus is an important parameter in models of population genetics as it permits one to estimate the timing of evolutionary divergence between species [1,6], and the effective population size of the species [7,8]. Mutation rates of microsatellites have long been estimated in numerous animal studies. One of the most important observations was that the mutation rate largely varies in several orders of magnitude among different species, ranging from 5 × 10^-6 ^in Drosophila [9-11] to 10^-3 ^in humans [12,13]. Clearly, the repeat motif length may influence microsatellite mutation rates, but the controversial has lasted many years. For example, Weber and Wong initially surveyed di- and tetranucleotide repeat microsatellites and estimated higher mutation rates for tetranucleotide repeats [14]. However, Chakraborty and his colleagues employed a method based on population variation to estimate relative differences in mutation rate and claimed that di- nucleotide repeats mutate at a rate 1.2–2.4 times higher than that of tri- or tetranucleotide repeats [15]. The finding has been confirmed by the latter studies [9,10,16,17]. These preliminary results showed that mutational dynamics at microsatellite loci could be more complicated than previously observed and assumed, at least at the interspecific level. Therefore, a picture of evolutionary dynamics of mutation rates among microsatellite loci of different repeat types at the genome-wide level is required for further understanding evolutionary factors which govern microsatellite variability and thus contributing to overall levels of genomic diversity across species. In this regard, knowledge of mutation dynamics is particularly necessary in plants, which have been insufficiently explored. Mutation rates at microsatellites in plants seem to be higher than in animals based on different levels of genetic diversity [18] as well as obviously limited experimental estimates [8,19,20].

The proper estimation of mutation rate at each locus is required to examine the variation of mutation rate among loci. Mutation rate of a microsatellite locus can be estimated from the direct genotype data [8,14,21]. Direct observation of such mutational events is preferable if possible, but involves much more time-consuming genotyping. It is quite costly in practice because mutation rates at most microsatellite loci are not large enough to be accurately estimated with a reasonable sample size. These assayed loci may not have sufficient representation of the genome-wide coverage of all microsatellites. Because of the above-mentioned limitations, it is impractical to study the mutation rate variation at the genome level using such a direct approach. Alternatively, the scaled mutation rate can be estimated using population genetic methods that utilize allelic frequency at large microsatellite loci in population samples. In such an analysis, it is commonly assumed that the population has reached a mutation-drift equilibrium so that the allele frequency distribution can be expressed in terms of the composite population parameter θ = 4Nμ, where N is the effective population size and μ is the mutation rate per locus per generation. Using this approach, for example, Xu and Fu recently developed an estimator of θ based on the genetic variation data at the microsatellite loci [22]. Their estimator is unbiased under the single-step stepwise mutation model and is robust against other forms of stepwise mutation models. It also has the advantage of being simple to compute and performs better than several existing estimators, including the maximum-likelihood-based estimator [23]. Taking advantage of this novel development, they further carried out an analysis of population mutation rate variation at di- nucleotide microsatellite loci in the human genome and demonstrated that their estimator is a reasonable assumption for the analysis of large genomic data [24].

**Figure 1:**
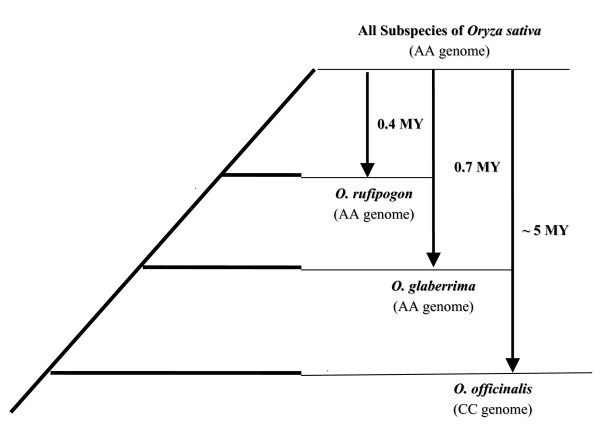
Species tree of the studied rice species [32].

As an important model crop, Asian cultivated rice (*Oryza sativa *L.) has two fully sequenced genomes [25,26]. With increasing population genetic data derived from genome-wide microsatellite loci [27-30], rice affords unique opportunities to use the above-mentioned population genetic method [22] to study their patterns of mutation rate. The increasing evidence [31-33] suggested that the majority of rice cultivars can be classified as two primary subspecies, *indica *and *japonica*, which were separated from their common wild progenitor *O. rufipogon *Griff. about 0.4 MYA (Million Years Ago) [34] (Figure 1). Genetic structure of this domesticated crop is so heterogenous that isozyme loci even distinguished additional population structure consisting of the six variety groups of *indica*, *japonica*, *aus*, *aromatic*, *rayada*, and *ashina *[35]. More recently, using 169 nuclear microsatellites and two chloroplast loci, five distinct groups were further confirmed within *O. sativa*, corresponding to *indica*, *aus*, *aromatic*, *temperate japonica*, and *tropical japonica *rices [29]. With regard to the subject of this work, there has been little comparative study on genome-wide patterns of mutation rate variation at microsatellites among closely related plant species and/or subspecies. Therefore, a variety of rice subspecies and their differently diverged relatives could be used as a valuable study model to gain genome-wide insights into patterns of mutation rates at microsatellite loci.

Variation in substitution rates has long been observed for the nucleotide sequence data. Some substitution models which have been proposed to fit the distribution of substitution rates [36,37]. Of them, gammar-distribution rates [38-41] are the most adopted ones. Recently, genome-wide analyses suggested that the gammar-distribution fits the population mutation rate distribution for the microsatellite loci in human [24]. It is of great interest to examine whether the mutation models will also fit the population mutation rate distribution for the microsatellite loci in rice and other related plant genomes.

In this study, we carried out an analysis of the population mutation rate in rice subspecies and related species at di-, tri-, and tetra-nucleotide microsatellite loci using three data sources. We collected and reported the first dataset of 60 microsatellite loci by genotyping Chinese representative samples which also included two related wild species, *O. rufipogon *and *O. officinalis *Wall. ex Watt., in addition to two Chinese subspecies (*indica *and *japonica*) of *O. sativa*. The second dataset we investigated is from a much more comprehensive dataset which were generated using a total of 169 microsatellites with world-wide sampling of different rice subspecies. Finally, we used the third dataset that was generated from a world-wide collection of the African cultivated rice *Oryza glaberrima *Steud., which diverged from the Asian cultivated rice about 0.7 MYA [34] (Figure 1), by assaying 93 microsatellite loci. Here, we reported the observed patterns of population mutation rate variation in microsatellite loci across rice subspecies and related species, and then attempted to provide evolutionary insights where possible.

## Results

### The gamma distribution of population mutation rates at di-, tri-, and tetra-nucleotide microsatellite loci

**Figure 2:**
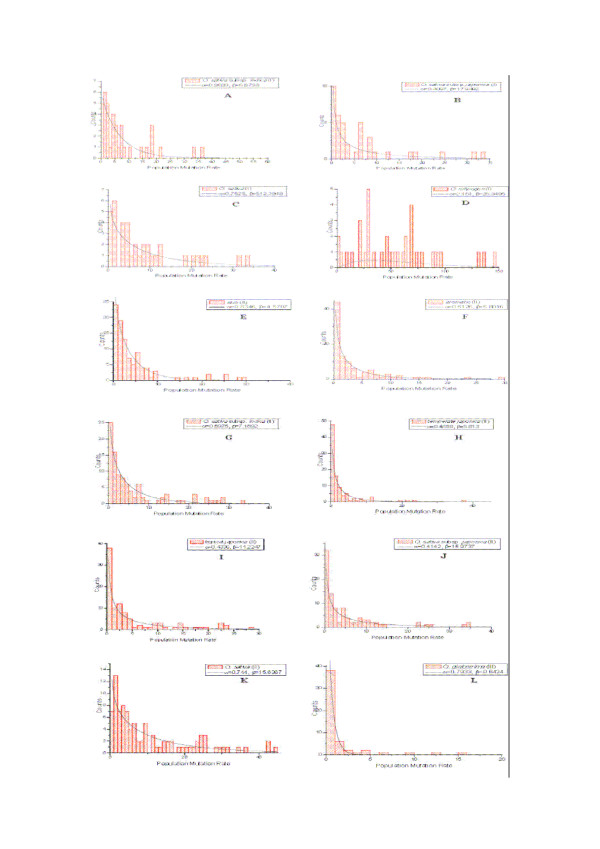
**Histogram of the population mutation rate and the fitted gamma distribution at di- nucleotide repeats in each of the studies species/subspecies**. A: *O. sativa *subsp. *indica *(I); B: *O. sativa *subsp. *japonica *(I); C: *O. sativa *(I); D: *O. rufipogon *(I); E: *aromatic *(II); F: *aus *(II); G: *O. sativa *subsp. *indica *(II); H: *temperate japonica *(II); I: *tropical japonica *(II); J: *O. sativa *subsp. *japonica *(II); K: *O. sativa *(II); L: *O. glaberrima *(III).

**Figure 3:**
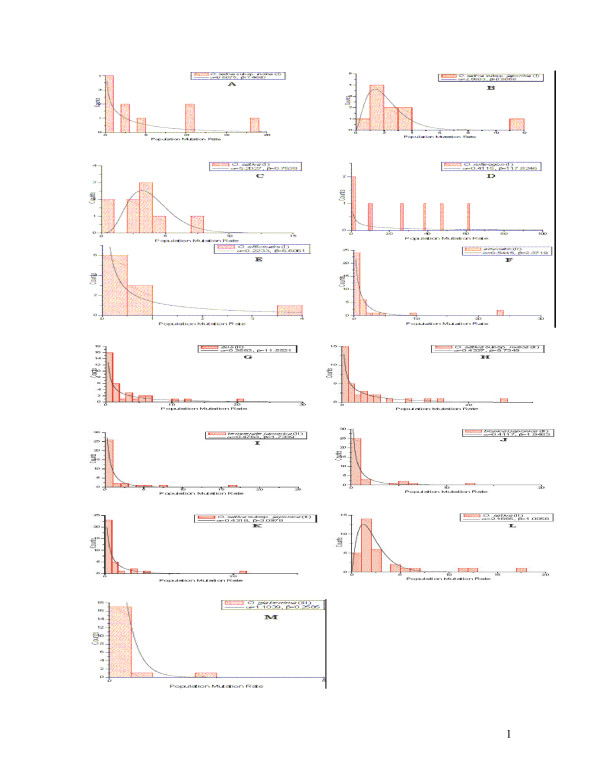
**Histogram of the population mutation rate and the fitted gamma distribution at tetra- nucleotide repeats in each of the studies species/subspecies**. A: *aromatic(II)*; B: *aus *(II); C: *O. sativa *subsp. *indica *(II); D: *temperate japonica *(II); E: *tropical japonica *(II); F: *O. sativa *subsp. *japonica *(II); G: *O. sativa *(II).

**Figure 4:**
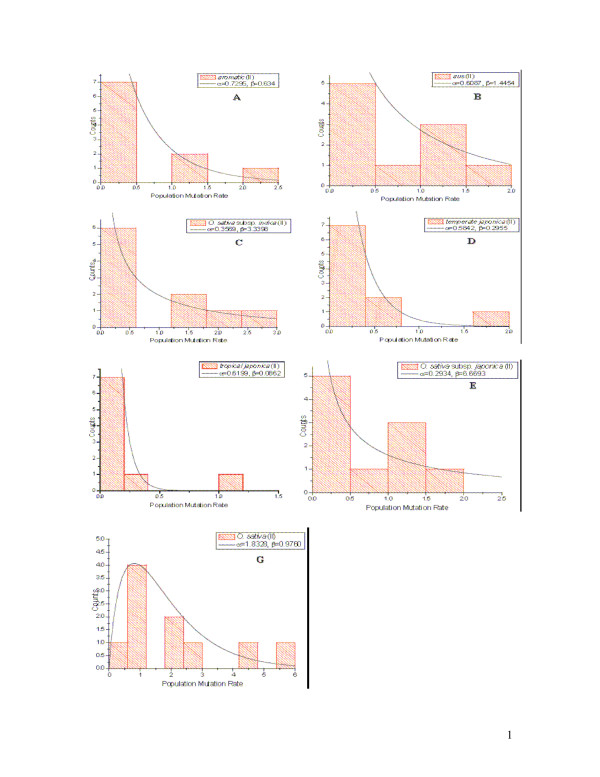
**Approximate posterior distribution of mutation rates (μ) at the analyzed microsatellite loci in *O. sativa *subsp. *indica *and *O. sativa *subsp. *japonica *using the rejection sampling procedure**. *O. sativa *subsp. *indica *was shown in red and *O. sativa *subsp. *japonica *was shown in blue.

The estimates of population mutation rate were computed for each locus in all rice species where genotypes are available. To examine variation in population mutation rate of microsatellites with respect to their motif sizes, we classified the analyzed loci into four types of repeats, namely di-, tri-, tetra-, and motif-complicated according to their motif sizes. Then the distribution of the population mutation rate in each category was plotted using histograms of population mutation rates at di-nucleotide repeats in all the species and/or subspecies studied. We found that the histograms can be fitted for most species and/or subspecies with a gamma distribution except *O. officinalis *(Figure 2). In the case of *O. officinalis*, we could not fit a gamma distribution probably due to missing data. However, the distributions of population mutation rates at microsatellites with tri-nucleotide repeats can also be approximated well with gamma distributions for all the species and/or subspecies without any exception (Figure 3). Because only Data set II has sufficient microsatellites with tetra-nucleotide repeats for *O. sativa *(Table 1), the histograms of population mutation rate can be fitted to the gamma distributions for the cultivated rice as well as all the included subspecies, respectively (Figure 4). A resampling based procedure was then performed to compare the estimates of population mutation rate at loci with different motifs from the same species and/or subspecies. Results show that the means of the population mutation rates at the loci with different motifs do not significantly differ at the 95% level in the studied rice species.

**Table 1:** Summary of the microsatellite loci and samples of the three datasets analyzed in the present study

Data set	Species/Subspecies/Group	Sample size	Repeat Types of Microsatellites	Loci Numbers
**Set I**	*O. sativa*	57	Total	60
	*O. sativa *subsp. *indica*	22	di-	39
	*O. sativa *subsp. *japonica*	35	tri-	10
	*O. rufipogon*	35	tetra-	0
	*O. officinalis*	21	motif-complicated	11
				
**Set II**	*O. sativa*	207	Total	169
	*O. sativa *subsp. *aromatic*	19	di-	99
	*O. sativa *subsp. *aus*	20	tri-	37
	*O. sativa *subsp. *indica*	79	tetra-	10
	*O. sativa *subsp. *japonica*	89	motif-complicated	23
	*temperate japonica*	45		
	*tropical japonica*	44		
				
**Set III**	*O. glaberrima*	198	Total	93
				
			di-	54
			tri-	20
			tetra-	2
			motif-complicated	17

### Dynamics of population mutation rates across related rice species

Our results show that population mutation rates largely vary among the four studied species (Table 2). Of them, *O. rufipogon*, the wild progenitor of *O. sativa*, possesses the largest mean population mutation rate, while *O. officinalis *exhibits the lowest value. In comparison, two cultivated rice apparently differ in population mutation rates. African cultivated rice *O. glaberrima *has at least fourfold lower value than Asian cultivated rice *O. sativa*. The interspecific comparisons at all the microsatellites further suggest that the two estimated parameters (both ***α ***and ***β ***) of a gamma distribution are the largest for *O. rufipogon *among all the three rice species (*O. rufipogon*, *O. sativa*, and *O. glaberrima*), which fit gamma distributions (Table 2). Interestingly, African cultivated rice *O. glaberrima *exhibited a slightly larger shape parameter ***α ***but a rather smaller scale parameter ***β ***than Asian cultivated rice *O. sativa *from both Data set I and II.

**Table 2:** Summary of the estimated θ values and parameters in a gamma distribution that fits the population mutation rate at all assayed microsatellites in each species and subspecies

Data set	Species/Subspecies	Mean	*α *(95%CI)	*β *(95%CI)
**Set I**	*O. sativa*	*7.2392*	0.7175 (0,6.3166)	9.5382 (3.0237,16.0527)
	*O. sativa *subsp. *indica*	*6.8069*	0.7219 (0,6.1893)	8.9435 (2.4755,15.4115)
	*O. sativa *subsp. *japonica*	*3.9607*	0.6157 (0,4.2666)	6.5953 (0.3233,12.8673)
	*O. rufipogon*	*56.2972*	1.1595 (0,13.8015)	46.3953 (43.5004,49.2902)
	*O. officinalis*	1.0156	N/A*	N/A*
				
**Set II**	*O. sativa*	*7.9047*	0.8256 (0,7.8399)	9.0353 (8.0004,10.0702)
	*O. sativa *subsp. *aromatic*	*2.0898*	0.5172 (0,7.6967)	4.1934 (2.0374,6.3494)
	*O. sativa *subsp. *aus*	*3.1579*	0.6709 (0,8.6246)	5.0972 (2.3924,7.8020)
	*O. sativa *subsp. *indica*	*4.3996*	0.5977 (0,4.1943)	7.2475 (6.3831,8.1119)
	*O. sativa *subsp. *japonica*	*4.6547*	0.3617 (0,5.3748)	12.5585 (11.5882,13.5287)
	*temperate japonica*	*1.8311*	0.4388 (0,7.0048)	4.2889 (1.6821,6.8957)
	*tropical japonica*	*3.7422*	0.3479 (0,7.8719)	10.3196 (7.1836,13.4556)
				
**Set III**	*O. glaberrima*	*0.3950*	0.9016 (0.0784,1.7248)	0.5019 (0,1.4466)

* N/A: cannot be fitted with a gamma distribution because of the failure of inter-specific amplification of microsatellites and thus extensively missing data

Patterns of population mutation rate variation were further investigated for the di-, tri-, and tetra-nucleotide motifs of the analyzed microsatellite loci, respectively (Table 3 and 4; Figure 4). A similar pattern was observed in the three species, *O. sativa*, *O. rufipogon *and *O. glaberrima*, at microsatellites with di-nucleotide repeats (Table 3). Although the estimator ***β ***exhibited a similar pattern in the above-mentioned species, at microsatellites with tri-nucleotide repeats as shown in Table 4, they somewhat had a reverse pattern for the parameter ***α; ***that is, *O. sativa *possessed a larger estimated value ***α ***than *O. rufipogon*. It is noticeable that one more species, *O. officinalis*, fits a gamma distribution at microsatellites with tri-nucleotide repeats. In comparison with the other three species, it had a small estimated ***α ***. It is clear, however, that *O. officinalis *had a larger ***β ***value than two cultivated rice *O. sativa *and *O. glaberrima*, although it still appears smaller than *O. rufipogon*.

**Table 3:** The estimates of parameters in a gamma distribution that fits the population mutation rate at di- nucleotide repeats in each species and subspecies

Data set	Species/Subspecies	*α *(95%CI)	*β *(95%CI)
**Set I**	*O. sativa*	0.7625 (0,3.6437)	12.3948 (8.9452,15.8444)
	*O. sativa *subsp. *indica*	0.9639 (0, 3.8119)	6.8738 (3.4242,10.3234)
	*O. sativa *subsp. *japonica*	0.4097 (0, 2.4461)	17.9492 (16.4792,19.4192)
	*O. rufipogon*	2.1610 (0.1761,4.1459)	28.3495 (27.2519,29.4471)
	*O. officinalis*	N/A*	N/A*
			
**Set II**	*O. sativa*	0.7440 (0, 7.2902)	15.6367 (14.1467,16.1267)
	*O. sativa *subsp. *aromatic*	0.5126 (0, 5.9990)	5.8016 (3.2340, 8.3692)
	*O. sativa *subsp. *aus*	0.8346 (0, 8.5119)	4.5707 (3.2379,5.9035)
	*O. sativa *subsp. *indica*	0.6975 (0, 4.5783)	7.1892 (5.0920,9.2864)
	*O. sativa *subsp. *japonica*	0.4142 (0, 6.5431)	15.9737 (15.6797,16.2677)
	*temperate japonica*	0.4518 (0, 8.0899)	5.813 (3.9314,7.6946)
	*tropical japonica*	0.4336 (0, 7.0976)	11.2247 (10.4407,12.0087)
			
**Set III**	*O. glaberrima*	0.7933 (0, 2.8709)	0.6424 (0, 2.034)

* N/A: see Table 2

**Table 4:** The estimates of parameters in a gamma distribution that fits the population mutation rate at tri- nucleotide repeats in each species and subspecies

Data set	Species/Subspecies	*α *(95%CI)	*β *(95%CI)
**Set I**	*O. sativa*	5.2027 (1.0867,9.3187)	0.7538 (0,3.1254)
	*O. sativa *subsp. *indica*	0.6075 (0, 10.1723)	7.4693 (5.8621,9.0765)
	*O. sativa *subsp. *japonica*	2.6803 (0, 8.3251)	0.8068 (0, 3.1980)
	*O. rufipogon*	0.4115 (0, 29.7135)	117.8246 (117.4381,118.2111)
	*O. officinalis*	0.2233 (0, 10.9641)	6.6061 (5.6065,7.6057)
			
**Set II**	*O. sativa*	2.1665 (0, 6.5177)	1.0056 (0, 7.1208)
	*O. sativa *subsp. *aromatic*	0.5445 (0, 2.5241)	2.3719 (1.2351,3.5087)
	*O. sativa *subsp. *aus*	0.3683 (0, 3.0143)	11.5521 (8.2005, 14.9037)
	*O. sativa *subsp. *indica*	0.4337 (0, 10.9393)	9.7349 (8.3982,11.0716)
	*O. sativa *subsp. *japonica*	0.4318 (0,7.0370)	3.0978 (0, 9.7226)
	*temperate japonica*	0.4763 (0, 6.8763)	1.7339 (0, 6.8299)
	*tropical japonica*	0.4117 (0, 3.1326)	1.9463 (0, 9.1983)
			
**Set III**	*O. glaberrima*	1.1039 (0, 2.2498)	0.2585 (0, 3.8767)

### Difference of population mutation rates among subspecies of Asian cultivated rice

In this study, population mutation rate variation of *O. sativa *was estimated by using two data sources. The Data set I was collected by using microsatellites and samples both fewer than Data set II [29]. Comparisons of parameters (***α ***and ***β ***) of the fitted gamma distributions show consistent estimates of population mutation rate variation from these two data sets (Table 2). Similar results could also be observed at microsatellites with di- and tri- nucleotide repeats from Data set I and II (Table 3, 4).

To explore whether there was any intra-specific difference in the population mutation rates of microsatellites at the genomic level, estimates of ***α ***and ***β ***of the gamma distribution were compared among the studied subspecies of *O. sativa *(Table 2). Analyses of the data sets show that subsp. *indica *possessed larger ***α ***estimate than subsp. *japonica*. Results from Data set II further indicates that the ***α ***estimate is the highest for subsp. *aus *while that for subsp. *japonica *is the lowest. Nevertheless, estimates for the shape parameter ***α ***do not differ significantly from each other, indicating that actual variation in mutation rates among the subspecies of *O. sativa *may not be apparent (Table 2, 3, and 4; Figure 4). It is clear that the scale parameter ***β ***has a very large variance among the estimates relative to the mean value ***α ***for the studied subspecies. Interestingly, *tropical japonica *had a larger ***β ***value than *temperate japonica *despite their very close genetic proximity.

**Figure 5:**
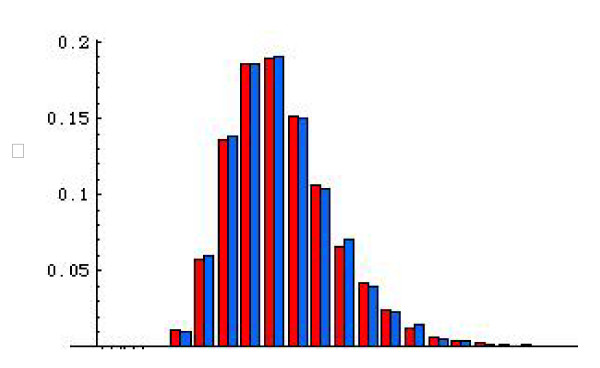
**Histogram of the population mutation rate and the fitted gamma distribution at tri- nucleotide repeats in each of the studies species/subspecies**. A: *O. sativa *subsp. *indica *(I); B: *O. sativa *subsp. *japonica *(I); C: *O. sativa *(I); D: *O. rufipogon *(I); E: *O. officinalis *(I); F: *aromatic *(II); G: *aus *(II); H: *O. sativa *subsp. *indica *(II); I: *temperate japonica *(II); J: *tropical japonica *(II); K: *O. sativa *subsp. *japonica *(II); L: *O. sativa *(II); M: *O. glaberrima *(III).

To further confirm the potential difference of mutation rates among subspecies described above, we estimated and compared mutation rates for the analyzed microsatellites in two major subspecies, *indica *and *japonica*, through coalescent simulations for Data set I (methods see [42]). As shown in Figure 5, we again found no obvious difference of mutation rates between them, suggesting that indeed these two subspecies may not have obtained obvious intra-specific difference of mutation rates at microsatellite loci since their domestication.

## Discussion

Using the genetic variation data at microsatellties throughout the genome, we estimated the population mutation rate ***θ ***at these loci in a total of four *Oryza *species. The estimator of ***θ ***was developed with the assumption of mutation-drift equilibrium and no assortative mating in the studied population [22]. While the samples of two cultivated rice in this study may reach mutation-drift equilibrium, it was estimated that, for example, the outcrossing rate is only 0–5 % in Asian cultivated rice *O. sativa *[43]. However, we would argue that the estimator of ***θ ***is still applicable to our data sets due to the following reasons. First, it may be true that the selfing rate is high for a particular population (e.g., a variety) of cultivated rice. In our study, only one individual is taken from each accession and the estimation can be performed after adjustment for population size; and second, the estimator is based on sample homozygosity, which is computed from allele frequency as given in Equation 1. The main effect of selfing is on the frequencies of genotypes rather than the allele frequencies. Therefore, the low outcrossing rate in selfing species such as *O. sativa *and *O. glaberrima *could have minimal effects on the estimator.

It is well recognized that the cultivated plants always had a complicated history of evolution and domestication. As a result, in this case of cultivated rice, there was an obvious population size expansion. However, since it is generally believed that on average the effective population size is constant for all the loci in a specific sample, our comparisons of population mutation rate within samples/species should be valid. But the comparison across species would be problematic. Therefore, we can only compare the α estimates based on the property of gamma distribution when making comparisons among different species.

It should be noted that the approach we take in our study does have limitations. Because we are estimating the population mutation rate θ, the variation in this parameter reflects both the variation of the underlying mutation rate and the variation of coalescent time at each locus, which could be very large. Only on average does the estimates of θ reflect the average mutation rate. Therefore, even though our approach does not allow the direct comparison of the exact distribution of the mutation rate, it allows us to make comparisons of the average mutation rate between the microsatellites with different repeat motifs, which is the main purpose of our study.

It is noticeable that Data set II was collected by using the world-wide samples of *O. sativa *which included almost all genetic or ecological subspecies [29]. A total of 146 microsatellites were sampled from the whole rice genome. In comparison, Data set I only used the representative samples from China by assaying relatively fewer microsatellites of the genome. However, comparisons of parameters (α and β) of a gamma distribution show relatively consistent estimates of population mutation rate variation in *O. sativa *using all the loci and the microsatellites with di-nucleotide repeats from these two data sets. This suggests that the sample size and number of microsatellties from Data set I may be sufficient to capture the mutation rate variation of the major class of microsatellites at the genomic level. However, since most of the microsatellites used in our study are di-nucleotide repeats and we have much fewer tri-nucleotide repeat loci (10 for Data set I but 37 for Data set II), it is not surprising to observe that the parameter estimates at the tri-nucleotide repeats in *O. sativa *from the two data sets are inconsistent. Noticeably, the ascertainment bias should be taken into consideration for each dataset under investigation when making comparisons among species and/or datasets. As the microsatellite loci were mainly chosen for a purpose of the detection of polymorphisms, the microsatellites used for genotyping were most likely not random, leading to an important bias about the distribution of theta values. For example, loci with low underlying theta values are very likely to be discarded earlier, because they were considered monomorphic. Therefore, it may be necessary to analyze microsatellite data based on the randomly selected loci and retest the patterns observed in this study.

As a main purpose of this work, it is of great interest to examine whether there is any variation of mutation rates across microsatellites of different repeat types at a genome-wide level among different subspecies of the same species, *O. sativa*, and among these closely related *Oryza *species. In contrast to the findings from humans [15-17,44], our observations indicate that the mean population mutation rate of microsatellites with different motifs does not make significant differences among subspecies of *O. sativa *or within this species. The same is true for African cultivated rice *O. glaberrima*, and the other two wild rice species, *O. rufipogon *and *O. officinalis*. The effective population size for the sample from the same species or subspecies is generally assumed to be same across the microsatellite loci. Therefore, our results suggest that the mean population mutation rates of microsatellites with different motifs do not make significant differences within the studied *Oryza *species or each subspecies of cultivated rice.

Our results suggest that the variation of population mutation rate at microsatellites within species can be approximated with a gamma distribution for the most studied species. In such a study to first investigate evolutionary dynamics of population mutation rates at microsatellites in Asian cultivated rice and its relatives, the gamma distribution was applied. In addition, the results show that the subspecies of *O. sativa *have similar α values. Since α is generally interpreted as the shape parameter of a gamma distribution, our results reveal that the shapes of the distributions of population mutation rate at microsatellites in the subspecies of *O. sativa *are similar. This conclusion has further been demonstrated through another separate coalescent simulation for Data set I. An easy interpretation is that these subspecies of *O. sativa *have not been separated long enough to cause any differences in the mutation rate at microsatellites. However, a very large variance of the scale parameter β was found among the studied subspecies. Such a difference in the distributions mainly comes from the different effective population sizes of these subspecies. From the viewpoint of the domestication and history of cultivation of this cultivated rice [29,32,33,35,43,45,46], it is possible that these genetic or ecological subspecies have different effective population size. Such a difference has contributed to the variability in the scale parameter β observed.

In contrast to consistent variation of population mutation rates at microsatellites among different subspecies within *O. sativa*, we observed apparent variation among the studied rice species. Both estimated parameters α and β of a gamma distribution are larger for the wild progenitor *O. rufipogon *than those for the derived cultivated rice *O. sativa*. Theoretically, it is a well-known fact that the wild progenitor always has a large effective population size. Therefore, this observation strongly suggests that the population mutation rates of microsatellites in cultivated rice have become small after its origin and domestication. The other African cultivated rice species, *O. glaberrima*, which was originated and is merely grown in Africa [47,48], exhibited a larger shape parameter α but a smaller scale parameter β than Asian cultivated rice *O. sativa*. This indicates that African cultivated rice may have a relatively high mutation rate of microsatellites. The result may also be indicative of small effective population size in *O. glaberrima*, which seems consistent to the origin and very narrow geographical range of this cultivar in Africa [47,48]. The most distantly related species *O. officinalis *fits a gamma distribution only at microsatellites with tri-nucleotide repeats, which showed a smaller α value even than *O. sativa*. In comparison, it had a smaller estimated α than any other species.

## Conclusion

In this study, genome-wide microsatellite data were analyzed for four *Oryza *species. It suggests that the population mutation rates at the assayed loci could be approximately fitted with a gamma distribution in most of the studied species. In addition, we obtained the shape parameters of their distributions. The results demonstrate that population mutation rate (***θ***) is able to efficiently estimate the genome-wide variation of mutation rates at microsatellite loci in plants. The results may help to guide the modeling of genome-wide genetic variability at microsatellite loci with different repeat motifs.

Our comparative analysis shows that the mean population mutation rate of microsatellites with different motifs does not make significant differences within the studied *Oryza *species or each subspecies of cultivated rice *O. sativa*. This finding is not consistent to previous results reported in other organisms (e.g., humans), although the inconsistence may come from the dramatically different sample sizes in these studies. More data from other plant taxa may help to further clarify our obtained result. Meanwhile, precise estimates of mutation rates of microsatellites with different motifs may be needed through collecting direct genotype data in closely related rice species.

Our data showed that population mutation rates largely vary across the rice species in this study. Such observations are indicative of the dynamics of mutation rates among them. It is also likely that they may possess different effective population size after the divergence from the common ancestor. Different evolutionary histories along these lineages may be used to explain the variation of mutation rates. To better elucidate patterns of mutation rate and affected factors, it is of great interest to further study and compare extensive taxonomic groups of the genus with different population genetic histories.

We found that different subspecies of *O. sativa *have similar shape parameters of the gamma distribution. The coalescent simulation also indicates that the variation of mutation rates among these subspecies of *O. sativa *is not apparent due to their short duration of divergence. However, both estimated parameters ***α ***and ***β ***of a gamma distribution are larger for the wild progenitor *O. rufipogon *than those for the derived subspecies of cultivated rice *O. sativa*. In addition to a decreased effective population size during the process of domestication of cultivated rice, it is likely that mutation rates of microsatellites in rice may have become small in comparison to wild progenitor. Accurate estimate of mutation rates in these subspecies of Asian cultivated rice as well as the wild progenitor in experimental populations may help to better investigate the dynamics of their effective population size. With such an effort, we will be able to deeply obtain evolutionary insights into mutation and evolution of microsatellite loci in cultivated rice species and wild relatives.

## Methods

### Data

#### Data set I

The first dataset consists of represents by genotyping a total of 113 accessions of cultivated rice and closely related species at 60 microsatellite loci (Table 1). These samples represent their entire geographical range of China and included 22 accessions of *O. sativa *subsp. *indica*, 35 accessions of *O. sativa *subsp. *japonica*, 35 accessions of *O. rufipogon*, and 21 accessions of *O. officinalis*. For each accession, one to five individuals were assayed. Information of the study accessions (accession name, accession number, geographical sources) was published elsewhere [49]. When this study was conducted in 1997, there were a total of 323 microsatellite loci publicly available [50-53]. We selected five loci for each chromosome totaling 60 primer pairs that are randomly distributed throughout the rice genome [30]. These microsatellites included 39 di-nucleotide, 10 trinucleotide, and 11 motif-complicated nucleotide. Microsatellite polymorphisms were assayed by specific PCR conditions following Panaud et al. (1996) [51]. PCR products were run on 6 % polyacrylamide denaturing gels and marker bands were revealed using the silver staining as described by Panaud et al. (1996) [51]. The null alleles were confirmed after several repetitions with different amplification conditions to ensure that no reaction failure existed. To determine the allele size, the samples were directly compared with band sizes from an allelic ladder prepared by amplification of an artificial mixture of DNA from all the assayed samples.

#### Data set II

The second data set was obtained from Dr. Susan McCouch's laboratory at Cornell University. It contains genotype data on 169 microsatellite loci, grouped by five major populations, *indica*, *aus*, *aromatic*, *temperate japonica*, and *tropical japonica*. These microsatellites included 99 di-nucleotide, 37 trinucleotide, 10 tetranucleotide, and 23 motif-complicated nucleotide (Table 1). The sample sizes are 79, 20, 19, 45, and 44, respectively (Table 1). Detailed information of microsatellite loci and the study samples were reported by Garris et al. (2005) [29].

#### Data set III

The third data set was also obtained from Dr. Susan McCouch's laboratory at Cornell University. It consists of genotype data at 93 microsatellite loci in *O. glaberrima*. These microsatellites included 54 di-nucleotide, 20 trinucleotide, 2 tetranucleotide, and 17 motif-complicated nucleotide (Table 1). The sample size for this data set is 198 (Table 1). Detailed information of microsatellite loci and the study samples were reported by Semon et al. (2005) [48].

### Statistical Methods

The estimates of population mutation rate and patterns of mutation rate variation in each subspecies/species were obtained by generally using the approach by Xu, Chakraborty, and Fu (2005) [24]. In brief, the analysis includes the following steps.

#### Estimation of population mutation rate

The allele frequency of each locus in each subspecies/species was estimated using the genotype data by the gene-counting method. We further estimated the composite parameter – population mutation rate ***θ = 4Nμ*, **where N is the effective population size and ***μ ***is the mutation rate per locus per generation. The parameter is critical in making inferences using genetic variations because many statistical properties of measures of genetic variation are dependent on the parameter. We estimated ***θ ***using the sample homozygoisty-based estimator developed by Xu and Fu (2004) [22], which is approximately unbiased under the single-step stepwise mutation model and has a much smaller variance than the size-variance based estimator. The estimator is also simple to compute, which makes it suitable for large-scale analysis of microsatellites at the genomic level.

The sample homozygosity is computed as

(1) $$\hat{F}=\left(n\sum_{i=1}^{k}p_{i}^{2}-1\right)/\left(n-1\right),$$

where *n *is the sample size, *k *is the number of alleles in the sample, and *p*_*i *_is the allele frequency estimate for the *i*th allele in the sample. A biased estimator is given by

(2) $${\tilde{\theta }}_{F}=\frac{1}{2}\left(\frac{1}{F^{2}}-1\right).$$

Then an unbiased estimate of ***θ ***is obtained through solving the corresponding equation for ***θ ***depending on the biased estimator ${\tilde{\theta }}_{F}$. For ${\tilde{\theta }}_{F}$ ≤ 15.0,

$${\tilde{\theta }}_{F}=\left(1.1313+\frac{3.4882}{n}+\frac{28.2878}{n^{2}}\right)\theta +0.3998\sqrt{\theta .}$$

For ${\tilde{\theta }}_{F}$ > 15.0,

$${\tilde{\theta }}_{F}=\left(1.1675+\frac{3.3232}{n}+\frac{63.698}{n^{2}}\right)\theta +0.2569\sqrt{\theta .}$$

The population mutation rate ***θ ***was estimated for each locus in every possible subspecies/species where allele frequency can be estimated from the genotype data.

#### Fitting with a gamma distribution

Previous studies have shown that the variation of mutation rate at the protein-enzyme loci and single-nucleotide sites can be approximated as a gamma distribution. A recent study also suggests that the gamma distribution can be used to model the mutation rate variation at microsatellite loci with di-nucleotide repeats in humans [24]. The exact pattern of mutation rate variation in rice is unknown. From the estimates of the population mutation rate for the microsatellites across the rice genome, we plotted the histogram, which shows that the distribution of the mutation rates in rice can be approximated with a gamma distribution. A maximum-likelihood approach was used to fit the histogram with a gamma distribution and to estimate the two parameters of the gamma distribution – shape parameter ***α ***and scale parameter ***β ***. The gamma distribution was parameterized such that the expectation is ***αβ ***and variance is ***αβ ***^2^. The estimates of the parameters from each subspecies were compared.

#### Comparing the θ Estimates

To compare the ***θ ***estimates from microsatellites with different motifs, we used a resampling procedure. Suppose we want to compare the ***θ ***estimates from di-nucleotide markers to those from tri-nucleotide markers. Suppose we had n tri-nucleotide markers, we re-sample 1,000 times with replacement n markers from the group of di-nucleotide markers and calculated the average of ***θ ***estimates from each resampling. The number of times N the average value was less than the average value from the tri-nucleotide markers was used to calculate a *P *value as N/1000.

### Coalescent Methods

#### Demographic Model

The population model of domestication used here approximates the demography of a domestication event of a single cultivated species from its wild progenitor (see [42] for details). It is assumed that the wild progenitor species has a constant-size population with size *N*2. The domestication begins at time *Td *in a small founder population of the cultivated species, which is merely a subset of the members in the wild progenitor. The domestication is assumed to have occurred in this constant-size founder population with size *N*1. Usually, *N*1 could be much smaller than *N*2. When the domestication is complete at time *Te*, it is assumed that the population size changes to *N*0, which is generally much larger than *N*1, representing a rapid population expansion of the domesticated species. Let *T0 *and *T*1 be the lengths of time when the population sizes are *N*0 and *N*1, respectively. Thus, this simple domestication model has five above-described demographic parameters.

#### Simulating microsatellite polymorphisms

Under such a population model, patterns of microsatellite variation in the two species (one subspecies and its progenitor) were simulated. On a simulated genealogy from two subspecies with the shared ancestral population [42], random mutations were placed and the changes of repeat numbers at microsatellites were simulated. To approximate the mutation process in microsatellites, we used a generalized stepwise model, under which the change of the number of microsatellite repeats follows a symmetric geometric distribution with a parameter *p*. In this study, we considered five models with *p *= 1, 0.9, 0.8, 0.5, and 0, and the results for *p *= 0.8 are shown in this article because the results under the five models are very similar.

#### Rejection-sampling algorithm for estimating demographic parameters

A rejection-sampling algorithm was employed to obtain estimates of the demographic parameters and the mutation rate in the above-described model [42]. Because evaluating the full likelihood of multiple microsatellites may be computationally infeasible at present to the best of our knowledge, we summarized the data by two statistics, the average heterozygosity in wild (*HR*) and the average heterozygosity in cultivated rice (*HC*), which could be either *HI *or *HJ *for the analysis of subsp. *indica *and subsp. *japonica*, respectively. The procedure of our rejection-sampling algorithm to obtain a sample from the joint posterior distribution of the mutation rate is as follows: 1) simulate the mutation rate of microsatellites from the prior distributions. For the mutation rate (*u*), we used a uniform distribution from 0 to 2 _10^-4 ^when *p *= 0.8; 2) place random mutations and simulate patterns of microsatellite polymorphisms. To incorporate the variation in mutation rate across loci, the mutation rate at each locus is assumed to follow a Gamma distribution with mean *u *(determined at Step 1) and SD 0.7 × *u*. For each case, at least 5,000 accepted sets of parameters were collected.

## Authors' contributions

Li-zhi Gao has contributed to the whole study design, collection of microsatellite Data Set I, partial data analysis, the whole manuscript preparation and revisions; Hongyan Xu made contributions to the model application and statistical analysis, and he also partially worked on the preparation and revisions of the manuscript. Two authors read and approved the final manuscript.
